# Combined cervicosternotomy and cervicotomy for true retrosternal goiters: a surgical cohort study

**DOI:** 10.1007/s13304-021-01027-1

**Published:** 2021-03-29

**Authors:** S. Van Slycke, A.-S. Simons, K. Van Den Heede, P. Van Crombrugge, K. Tournoy, P. Simons, H. Vermeersch, N. Brusselaers

**Affiliations:** 1 Department of General and Endocrine Surgery, Onze-Lieve-Vrouw (OLV) Hospital Aalst, Moorselbaan 164, 9300 Aalst, Belgium; 2 Department of Head and Skin, University Hospital Ghent, Corneel Heymanslaan 10, 9000 Ghent, Belgium; 3 Department of General Surgery, AZ Damiaan, Gouwelozestraat 100, 8400 Ostend, Belgium; 4 Group of Biomedical Sciences, University Hospital Leuven, Herestraat 49, 3000 Leuven, Belgium; 5 Department of Endocrinology, Onze-Lieve-Vrouw (OLV) Hospital Aalst, Moorselbaan 164, 9300 Aalst, Belgium; 6 Department of Pneumology, Onze-Lieve-Vrouw (OLV) Hospital Aalst, Moorselbaan 164, 9300 Aalst, Belgium; 7 Department of Internal Medicine and Paediatrics, University Hospital Ghent, Corneel Heymanslaan 10, 9000 Ghent, Belgium; 8 Department of Radiology, Onze-Lieve-Vrouw (OLV) Hospital Aalst, Moorselbaan 164, 9300 Aalst, Belgium; 9 Plastic and Reconstructive Surgery, Department of Human Structure and Repair, University Hospital Ghent, Corneel Heymanslaan 10, 9000 Ghent, Belgium; 10 Department of Microbiology, Tumour and Cell Biology, Centre for Translational Microbiome Research, Karolinska Institutet, Karolinska Hospital, Tomtebodavagen 16, 17165 Stockholm, Sweden; 11 Global Health Institute, Antwerp University, Campus Drie Eiken, Gouverneur Kinsbergencentrum, Doornstraat 331, 2610 Wilrijk, Belgium

**Keywords:** Thyroid, Intrathoracic goiter, Surgery, Sternotomy, Morbidity, Hypocalcemia, Nerve paralysis

## Abstract

**Objective:**

Intrathoracic goiters are a heterogeneous group characterized by limited or extensive substernal extension. Whereas the former can be treated through cervicotomy, the latter sometimes requires a cervicosternotomy. Whether cervicosternotomy leads to more morbidity remains unclear.

This study aimed to compare intra- and postoperative morbidity in patients treated by cervicotomy or cervicosternotomy for intrathoracic goiters and standard thyroidectomy.

**Methods:**

In a prospectively gathered cohort undergoing thyroid surgery (2010–2019) intra- and postoperative morbidity of cervicotomy (*N* = 80) and cervicosternotomy (*N *= 15) for intrathoracic goiters was compared to each other and to a ‘standard’ thyroidectomy (*N* = 1500).

**Results:**

An intrathoracic extension prior to surgery was found in 95 (6%) of all thyroidectomies. Eighty patients (84%) were operated by cervicotomy and 15 (16%) by cervicosternotomy. The risk of temporary recurrent laryngeal nerve palsy was much higher in the cervicosternotomy group (21%) compared to cervicotomy (4%) and standard thyroidectomy (3%). The risk of temporary hypocalcemia after cervicotomy (28%) was comparable to a standard thyroidectomy (32%) but higher after cervicosternotomy (20%). No cases of permanent hypocalcemia or laryngeal nerve palsy were observed in both groups with substernal extension. The need for surgical reintervention was significantly higher in the cervicotomy group (6%) compared to cervicosternotomy (0%) and standard thyroidectomy (3%).

**Conclusion:**

In patients undergoing thyroid surgery for an intrathoracic goiter, cervicosternotomy was associated with more temporary laryngeal nerve palsy, but none of the interventions resulted in higher risks of permanent nerve damage, permanent hypocalcemia, or reintervention for bleeding. Reintervention was even more common after cervicotomy compared to cervicosternotomy.

**Level of evidence:**

IV

**Supplementary Information:**

The online version contains supplementary material available at 10.1007/s13304-021-01027-1.

## Introduction

Although there is no uniform definition of an intrathoracic goiter, many agree that a thyroid gland volume over 50% below the thoracic inlet is an important criterion [1–4]. “Retrosternal” and “substernal” goiters are synonyms. In the absence of a clear definition, the exact prevalence is difficult to determine. In patients undergoing thyroid surgery, the prevalence of intrathoracic goiters is approximately 1–21% [1, 2, 5].

In the case of an asymptomatic cervical goiter, conservative treatment may be followed [6]. In case of an asymptomatic, intrathoracic goiter two valid options are available: surgery or observation with monitoring. These options depend on the size of the substernal goiter and the patient characteristics (fit for surgery or not). Possible risks of a conservative policy consist of acute airway obstruction, bleeding, vena cava compression, malignancy and major surgery [7]. Suppressive hormonal therapy can reduce goiters in size, but in most cases, this will not be sufficient to alleviate compressive symptoms [6, 8].

The only successful surgical treatment for intrathoracic goiter is a total thyroidectomy with “en bloc” removal of the intrathoracic part, without capsular disruption [1, 6]. For this procedure, two different approaches are available: the cervical one through cervicotomy and the thoracic one through cervicosternotomy. In most cases, a cervical approach is sufficient to extract the mediastinal thyroid part by digital manoeuvres [1–6]. In more extensive cases a thoracic access is needed, in particular for goiters descending into the posterior mediastinum and below the aortic arch [1–3, 5] or with a diameter larger than the thoracic inlet [1, 5]. Less frequent are primary intrathoracic goiters, patients with severe kyphosis, superior vena cava syndrome, distortion of mediastinal structures and goiters with malignancy characteristics such as strong adhesions with neovascular capsule and microcalcifications [1, 4, 5].

This study aimed to assess intra- and postoperative morbidity in patients treated for intrathoracic goiter by cervicotomy or cervicosternotomy in comparison to a total surgical cohort of 1500 patients.

## Methods

### Study design

All consecutive adult patients undergoing thyroid surgery at the Onze Lieve Vrouw (OLV) hospital in Aalst, Belgium were enrolled in a prospective cohort to evaluate morbidity and outcome in a tertiary referral center. Recruitment of patients was started in January 2010 and is still ongoing. All data have been collected in a prospective manner, and all consecutive patients during the study were invited to participate (none declined consent). The cohort is registered at Research Registry (researchregistry6182) in accordance with the World Medical Association's Declaration of Helsinki 2013.

### Setting

All procedures were performed by a single, extensively trained endocrine surgeon (SVS), with a personal experience of over 5000 cases and an annual caseload of over 250 cases a year. The OLV Hospital in Aalst is the ninth largest general hospital in Belgium, with 844 recognized beds. It is a tertiary referral center for endocrine surgery. In 2018, almost 57,000 surgical interventions were performed and over 1 out of 30 thyroidectomies in Belgium is performed at this hospital.

### Ethics

Prospective data collection in these patients has been approved by the Ethics Committee of the OLV Hospital in Aalst, and all patients provided written informed consent.

### Study participants

The database was locked and ready for analysis on November 11th, 2019. In the overall cohort, all patients receiving surgery for intrathoracic goiter were selected and compared to the first 1500 consecutive patients operated for other thyroid conditions (referred to as ‘standard thyroidectomy’). The study cohort is outlined in supplementary Fig. 1. In the presence of a retrosternal goiter (on ultrasound or on clinical examination) a CT-scan was performed. The indication for surgery and choice of surgical technique was discussed at a multidisciplinary meeting, consisting of at least one radiologist, nuclear medicine specialist, endocrinologist, endocrine surgeon and pathologist. In case of a suspicious nodule on US, a fine needle aspiration was always recommended and performed to exclude a possible malignancy, regardless of the size of the thyroid or the possible retrosternal extent. More extended multidisciplinary planning was done for patients undergoing a possible cervicosternotomy, including a pulmonary check-up by an anesthesiologist and pneumologist, with lung function testing and bronchoscopy. The choice of the endotracheal tube size for anaesthesia was based on findings of the bronchoscopy and measurements of tracheal diameter on imaging. Pre-operative respiratory physiotherapy was foreseen to reduce the risk of pulmonary complications.

**Fig. 1:**
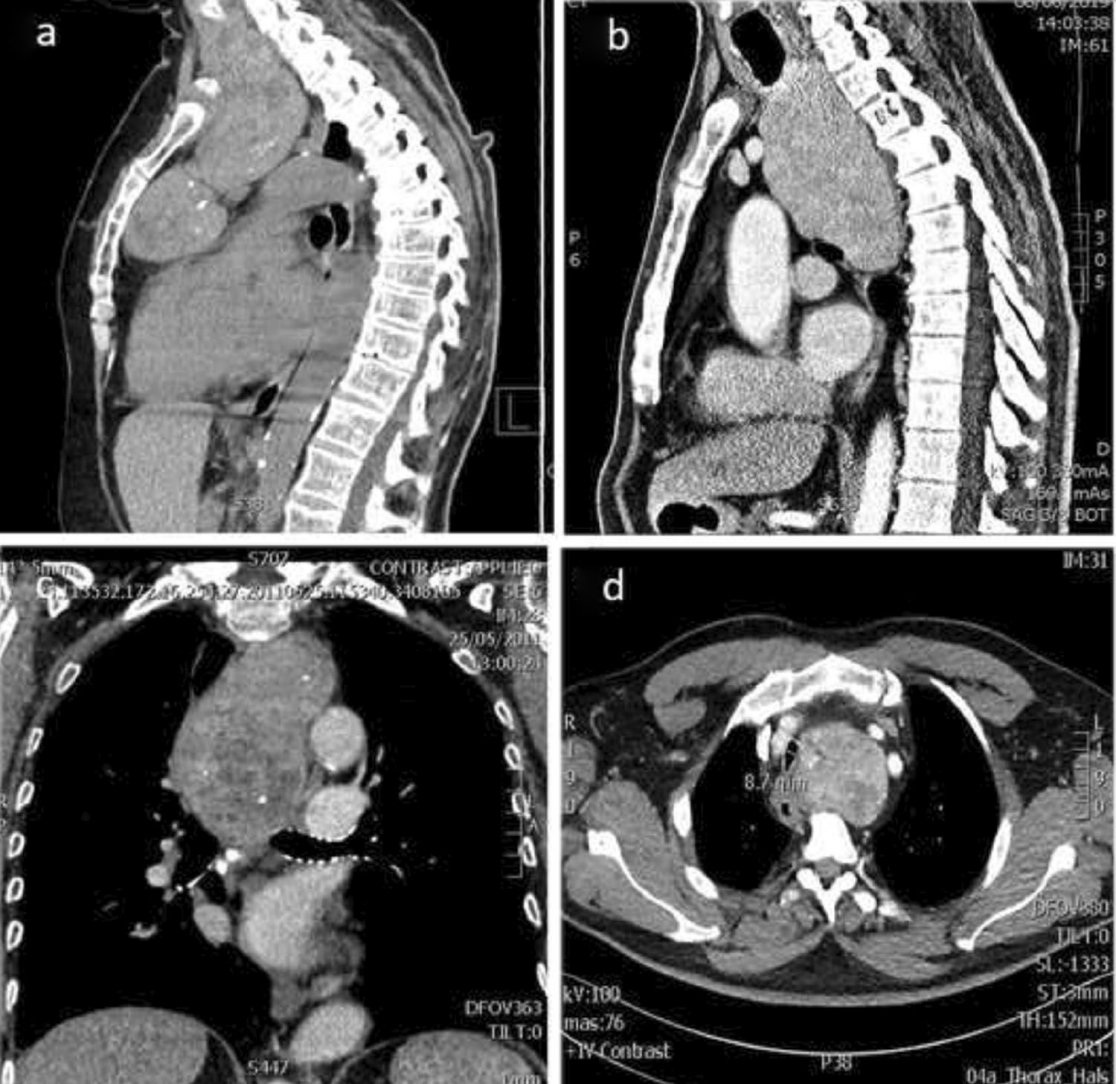
Preoperative Computed Tomography: intrathoracic goiter (**a**) in anterior mediastinum, (**b**) in posterior mediastinum, (**c**) extending below the carina (**d**) causing trachea deviation and narrowing

### Surgical characteristics

As proposed by the IONM study group guidelines, preoperative laryngoscopy was performed before surgery and IONM was used in every single case as part of our routine protocol.

Cervicosternotomy was used for goiters if 50% of the volume was located below the plane of the thoracic inlet and/or if an additional risk factor was present: wider than the thoracic inlet, diving below the aortic arch or in the posterior mediastinum. A cervicotomy was used for goiters without any of the above findings.

Surgery for an intrathoracic goiter always starts with a Kocher incision, exactly the same as the classic cervical approach. After identifying the recurrent laryngeal nerve, it is monitored and followed further down using the ‘toboggan technique’ as described by Charles Proye [10]. Intra-operative neuromonitoring during thyroid surgery is used in all cases to improve vigilance [11, 12]. It consists of an S-shaped probe for continuous vagal stimulation, a hand-held bipolar stimulator and acquisition electrodes as previously described [13, 14].

Based on the preoperative multidisciplinary discussion and the perioperative findings, a sternal split is performed when the intrathoracic part cannot be safely delivered.

### Definitions

In our series, the following definition of a substernal goiter was used consistently: more than 50% of the thyroid is located below the thoracic inlet. Patients selected for surgery for an intrathoracic goiter all received a pre-operative neck ultrasound, and an additional computed tomography (CT) scan or PET-CT scan to determine the optimal surgical technique [3, 9]. In case of a suspicious nodule on US, a fine needle aspiration was always recommended and performed, regardless the size of the thyroid or the possible retrosternal extent to exclude an underlying malignancy.

Permanent hypocalcemia was defined as hypocalcemia lasting at least 6 months after surgery (albumin-corrected calcium level < 2.00 mmol/l or the need for calcium and/or activated vitamin D substitution to maintain normal calcium levels).

Pre-operative laryngoscopy is performed before anesthetic induction of the patient. Post-operative laryngoscopy is performed within 24hrs after surgery. In case of a documented RLN palsy, the patient is routinely followed by the ENT and the surgical department until recovery or persistence. Temporary RLN palsy was defined as a recuperation of vocal cord mobility on laryngoscopy within one year after surgery. Permanent palsy was defined as absence of vocal cord mobility on laryngoscopy after a one-year follow-up.

### Outcome

For all patients, data were collected on age (categorized as < 40, 40–59 and ≥ 60 years), sex (men or women), body mass index (categorized as < 25, 25–29, and ≥ 30 kg/m2), period of surgery (categorized as 2010–2012, 2013–2015, 2016–2019) and health status as defined by the American Society of Anaesthesiologist association [ASA, categorized as I–II [healthy or mild], and III–IV (severe systemic disease)]. Surgical indications were categorized as multinodular goiter, Graves’ disease, solitary nodule, toxic adenoma, amiodarone-induced hyperthyreosis and cancer.

Primary outcome variables were: (1) hypocalcemia (transient and permanent), (2) recurrent laryngeal nerve (RLN) palsy (transient and permanent), (3) reintervention for bleeding, (4) Clavien score ≥ 3 (indicating surgical reintervention). Hypocalcemia was only assessed in individuals receiving a total or completion thyroidectomy and was defined as a parathyroid hormone (PTH) level of < 15 ng/l 4 h after surgery. RLN palsy was only assessed in individuals with normal results at the pre-operative vocal cord assessment (defined as a moving vocal cord on laryngoscopy, performed by an independent ENT specialist).

### Statistical analysis

Descriptive statistics were compared by means of the Chi-square test, with *p* values < 0.05 indicating statistically significant results.

All primary outcomes were assessed by means of logistic regression, presented as odds ratios (OR) and 95% confidence intervals and adjusted for age, sex, BMI, period of surgery, health status, indication for surgery and hemi- or total thyroidectomy. Data on other complications was also collected and described.

Indicators for surgical complexity were weight of the resected specimen (grams), blood loss (mL), duration of surgery (minutes) and length of hospital stay (days). all were presented as median with interquartile ranges (IQR). The indicators were compared between cervicosternotomy and cervicotomy, and standard thyroidectomy by means of the Mann–Whitney *U* test, with *p* values < 0.05 indicating statistically significant differences.

All statistical analyses were conducted in Stata MP14 (Stata Corporation^®^).

## Results

### Patient characteristics

In this cohort, 1595 patients underwent thyroid surgery. No intrathoracic extension was seen in 1500. These were all treated through cervicotomy. In 95 patients (6%) an intrathoracic goiter was present. Eighty patients had a limited intrathoracic goiter and were operated by cervicotomy. In 15 patients an extensive intrathoracic goiter was present, requiring a resection by cervicosternotomy. Clinical characteristics of the different cohorts are shown in Table 1. Patients undergoing cervicotomy and cervicosternotomy were significantly older than the reference standard thyroidectomy group. A higher proportion of patients were men. BMI and ASA grade were slightly higher among the cervicosternotomy and cervicotomy groups.

**Table 1:** Descriptive characteristics of all individuals operated by cervicosternotomy or cervicotomy for an intrathoracic goiter, compared to standard thyroidectomy

	Standard thyroidectomy (reference, *N* = 1500)	Cervicotomy (*N* = 80)		Cervicosternotomy (*N* = 15)	
	*N* (%)	*N* (%)	*p* value (Chi-square)	*N* (%)	*p* value (Chi-square)
Age (years)					
< 40	289 (19.3)	2 (2.5)	< *0.001*	0 (0.0)	< *0.001*
40–59	714 (47.6)	30 (37.5)		0 (0.0)	
≥ 60	497 (33.1)	48 (60.0)		15 (100)	
Sex					
Male	304 (20.3)	30 (37.5)	< *0.001*	10 (66.7)	< *0.001*
Female	1196 (79.7)	50 (62.5)		5 (33.3)	
Body mass index (kg/m^2^)					
< 25	690 (46.0)	24 (30.0)	*0.004*	4 (26.7)	*0.263*
25–29.9	522 (34.8)	30 (37.5)		8 (53.3)	
≥ 30	288 (19.2)	26 (32.5)		3 (20.0)	
ASA-score					
I (healthy) or II (mild)	1333 (88.9)	61 (74.2)	*0.001*	7 (46.7)	< *0.001*
III–IV (severe or life threatening)	167 (11.1)	19 (23.8)		8 (53.3)	
Pre-op vocal cord assessment					
Abnormal	19 (1.3)	1 (1.3)	*0.990*	1 (6.7)	*0.068*
Thyroidectomy					
Partial	457 (30.5)	12 (15.0)	*0.003*	5 (33.3)	*0.810*
Total	1043 (69.5)	68 (85.0)		10 (66.7)	
Indication					
Benign	1,408 (93.9)	78 (97.5)	*0.181*	14 (93.3)	*0.932*
Malign	92 (6.1)	2 (2.5)		1 (6.7)	
Indication benign					
Multinodular goiter	785 (52.3)	67 (83.8)	< *0.001*	14 (93.3)	*0.052*
Graves	158 (10.5)	2 (2.5)		0 (0.0)	
Solitary nodule	375 (25.0)	6 (7.5)		0 (0.0)	
Toxic adenoma	64 (4.3)	2 (2.5)		0 (0.0)	
Amiodarone	26 (1.7)	1 (1.3)		0 (0.0)	
Year of surgery					
2010–2012	515 (34.3)	3 (3.8)	< *0.001*	1 (6.7)	*0.006*
2013–2015	466 (31.1)	3 (3.8)		3 (20.0)	
2016–2019	519 (34.6)	74 (92.5)		11 (73.3)	

p-values < 0.05 are considered statistically significant

*ASA* American Society for Anaesthesiology

**Fig. 2:**
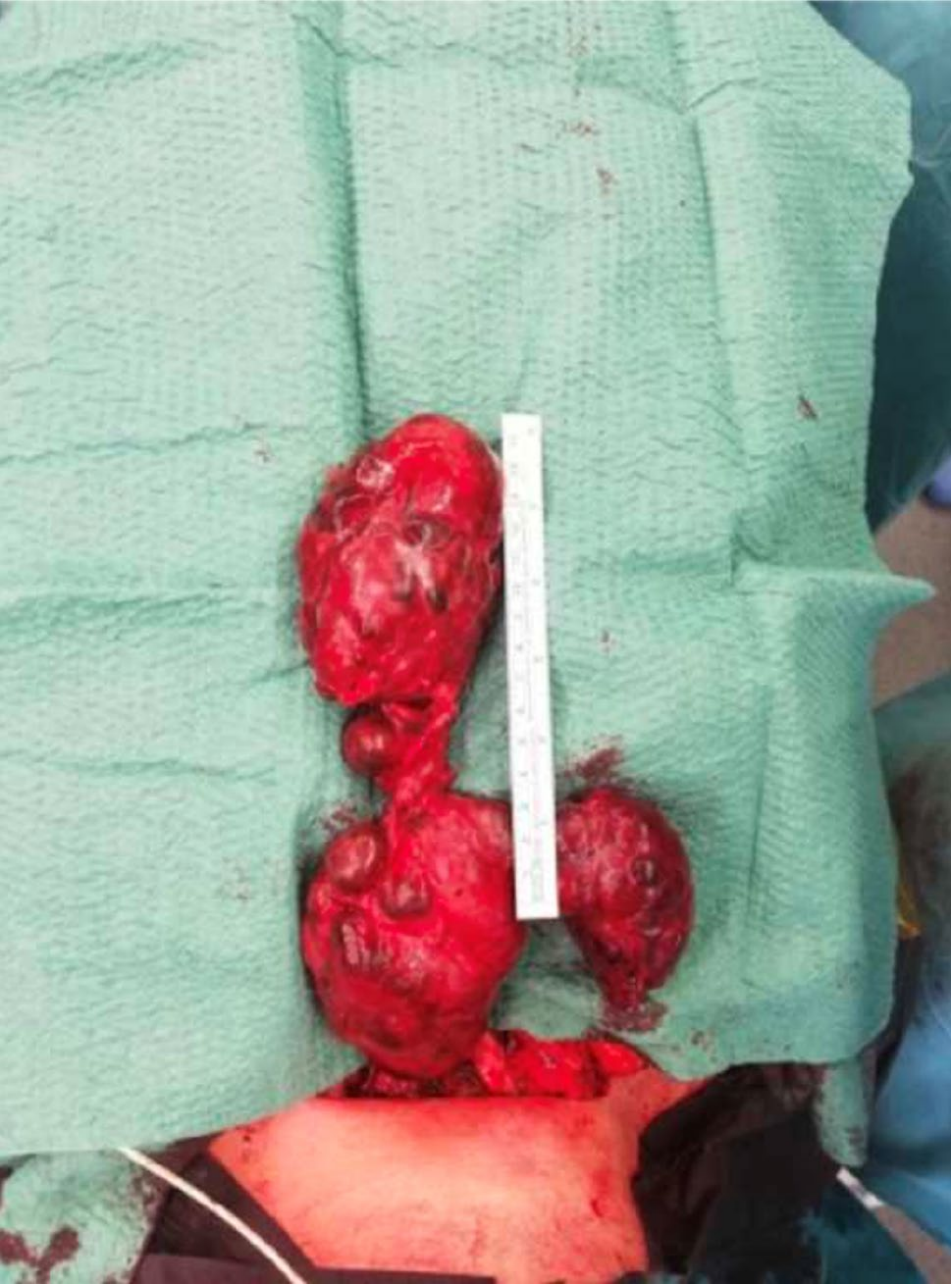
Specimen of an intrathoracic goiter, weighting 534 g, resected using the thoracic approach in one of the included patients

Clinical presentation of the 15 patients with extensive intrathoracic goiter is described below. Ten patients (67%) had compressive symptoms (superior vena cava syndrome, hoarseness or stridor). Four patients had an asymptomatic, but clinically visible goiter. One patient was diagnosed incidentally on a routine chest X-ray. The 15 extensive retrosternal goiters were located in the anterior (*N* = 4, Fig. 1a) and posterior mediastinum (*N* = 11, Fig. 1b). Twelve goiters reached below the aortic arch (Fig. 1c, Fig. 2). The first rib, the upper edge of the manubrium and the posterior edge of the first thoracic vertebrae were the landmarks to determine the thoracic inlet on CT-scan. Eight goiters had a larger diameter in the axial plane than the diameter of thoracic inlet, 13 goiters crossed the midline. One of the 15 goiters showed malignancy characteristics. There were no primary intrathoracic goiters. All 95 patients with a suspected retrosternal thyroid on US or on clinical examination underwent a CT scan. In the control group, an additional 365 patients underwent a CT scan for other reasons. Sixty-Eight percent of all 1595 patients underwent a scintigraphy. Only 69 patients in the whole cohort underwent a PET/CT.

### Primary outcomes

No cases of permanent hypocalcemia were recorded after total thyroidectomy by cervicosternotomy. However, 20%, 28% and 32% of cervicosternotomy, cervicotomy and standard thyroidectomy cases developed a transient hypocalcemia (Table 2a), however not resulting in statistically different risks after adjustment for confounding variables (Table 3).

**Table 2:** (a) Post-operative morbidity and (b) indicators of surgical complexity in all individuals operated by cervicosternotomy or cervicotomy for retrosternal goiters, compared to standard thyroidectomy

(a)					
Primary results	Standard thyroidectomy (reference) (*N* = 1500)	Cervicotomy (*N* = 80)		Cervicosternotomy (*N* = 15)	
	*N* (%)	*N* (%)	*p* value (Chi-square)	*N* (%)	*p* value (Chi-square)
Hypocalcemia (only in total thyroidectomy)					
All	365 (35.0)	19 (27.9)	*0.236*	2 (20.0)	*0.322*
Transient	333 (31.9)	19 (27.9)	*0.395*	2 (20.0)	*0.386*
Permanent	32 (3.1)	0 (0.0)	*0.129*	0 (0.0)	*0.539*
Recurrent laryngeal nerve palsy (only with normal preoperative vocal cords)					
All	65 (4.4)	3 (3.8)	*0.802*	3 (21.4)	*0.002*
Transient	41 (2.8)	3 (3.8)	*0.610*	3 (21.4)	< *0.001*
Permanent	24 (1.6)	0 (0.0)	*0.257*	(0.0)	*0.666*
Surgical reintervention for bleeding	39 (2.6)	5 (6.3)	*0.053*	0 (0.0)	*0.527*
Clavien score ≥ 3 (requiring surgical intervention)	39 (2.6)	2 (2.5)	*0.956*	0 (0.0)	*0.527*

(b)					
Indicators for surgical complexity	Standard thyroidectomy (reference) (*N *= 1500)	Cervicotomy (*N* = 80)		Cervicosternotomy (*N* = 15)	
	Median (interquartile range) [max]	Median (inter-quartile range) [max]	*p* value (Mann–Whitney *U*)	Median (inter-quartile range) [max]	*p* value (Mann–Whitney *U*)
Hemi-thyroidectomy					
Weight of resected specimen (grams)	26 (16–44) g	33 (20–70) g	*0.225*	355 (280–378) g	*0.003*
	[298 g]	[152 g]		[378 g]	
Length of stay (days)	1 (1–1) days	1 (1–1) days	*0.320*	7 (4–8) days	< *0.001*
	[15 days]	[1 day]		[8 days]	
Blood loss (mL)	6 (2–13) mL	10 (4–32) mL	*0.126*	374 (263–695) mL	< *0.001*
	[498 mL]	[600 mL]		[1000 mL]	
Duration of surgery (minutes)	40 (35–50) min	53 (40–70) min	*0.041*	140 (135–140) min	< *0.001*
	[275 min]	[90 min]		[300 min]	
Total thyroidectomy					
Weight of resected specimen (grams)	64 (36–112) g	123 (69–172) g	< *0.001*	430 (400–502) g	< *0.001*
	[848 g]	[528 g]		[776 g]	
Length of stay (days)	1 (1–1) days	1 (1–1) days	*0.763*	5 (1–7) days	< *0.001*
	[13 days]	[21 days]		[10 days]	
Blood loss (mL)	18 (9–38) mL	37 (14–71) mL	< *0.001*	350 (204–634) mL	< *0.001*
	[1290 mL]	[536 mL]		[1100 mL]	
Duration of surgery (minutes)	65 (55–85) min	73 (60–90) min	*0.029*	180 (150–200) min	< *0.001*
	[540 min]	[180 min]		[240 min]	

p-values < 0.05 are considered statistically significant

**Table 3:** The risk of hypocalcemia and recurrent laryngeal nerve palsy among individuals operated with cervicotomy or cervicosternotomy compared to standard thyroidectomy, by means of (multivariable) logistic regression

	Standard thyroidectomy (reference)	Cervicotomy (*N* = 80), odds ratio (95% CI)		Cervicosternotomy (*N* = 15), odds ratio (95% CI)	
		Crude	Adjusted^a^	Crude	Adjusted^a^
Hypocalcemia (transient or permanent)^b^	1.00	0.72 (0.42–1.24)	0.81 (0.46–1.45)	0.46 (0.10–2.20)	0.50 (0.10–2.46)
Recurrent laryngeal nerve palsy (transient or permanent)^c^	1.00	0.86 (0.26–2.80)	0.71 (0.21–2.43)	5.94 (1.62–21.81)	4.20 (1.03–17.07)
Surgical re-intervention for bleeding	1.00	2.50 (0.96–6.52)	1.56 (0.54–4.49)	–	–
Clavien score ≥ 3	1.00	0.96 (0.23–4.05)	0.63 (0.14–2.85)	–	–

*CI* confidence interval

^a^Adjusted for age, sex, body mass index, ASA score, hemi- or total thyroidectomy, period of surgery

^b^Only individuals receiving total thyroidectomy are included

^c^Only individuals without pre-operative abnormal vocal cord assessment

No cases of permanent RLN palsy were observed for all retrosternal goiters. Three cases of transient nerve palsy were observed in the cervicotomy and cervicosternotomy groups (Table 2a), resulting in an increased risk of RLN paralysis for cervicosternotomy compared to standard thyroidectomy after adjustment for confounding variables [OR = 4.20 (1.03–17.07)] (Table 3).

There were no surgical reinterventions for bleeding in the cervicosternotomy group, and only 5 (6%) and 39 (3%) in the cervicotomy and standard thyroidectomy groups (Table 2a). Differences were not statistically significant (Table 3).

Three patients of the cervicosternotomy group had pulmonary morbidity, including a post-sternotomy syndrome with persistent pleural effusion, a postoperative pneumonia and a right apical pneumothorax. None of the patients receiving cervicotomy or standard thyroidectomy had pulmonary complications (Fig. 2).

### Surgical complexity indicators

For hemithyroidectomies in the cervicotomy group, the duration of surgery was slightly longer compared to standard hemithyroidectomy (median 53 min compared to 40 min). For total thyroidectomies, the weight of the resected specimen (median 123 g versus 64 g), the amount of blood loss (37 mL versus 18 mL) and duration of surgery (73 min versus 65 min) were higher compared to the standard thyroidectomy group. In the cervicosternotomy group, the weight of the resected specimen and blood loss were higher, and the duration of surgery and length of stay longer compared to standard thyroidectomy, for both hemi-thyroidectomy and total thyroidectomy (Table 2b).

## Discussion

This surgical cohort of 1500 consecutive thyroid surgery procedures contains one of the largest series of retrosternal goiters ever reported. All procedures were carried out using a standardized approach and standardized definitions and recorded without missing data. This single centre, prospective surgical cohort showed a prevalence of 6% of intrathoracic goiter, comparable to other published data [2, 5, 15].

**Fig. 3:**
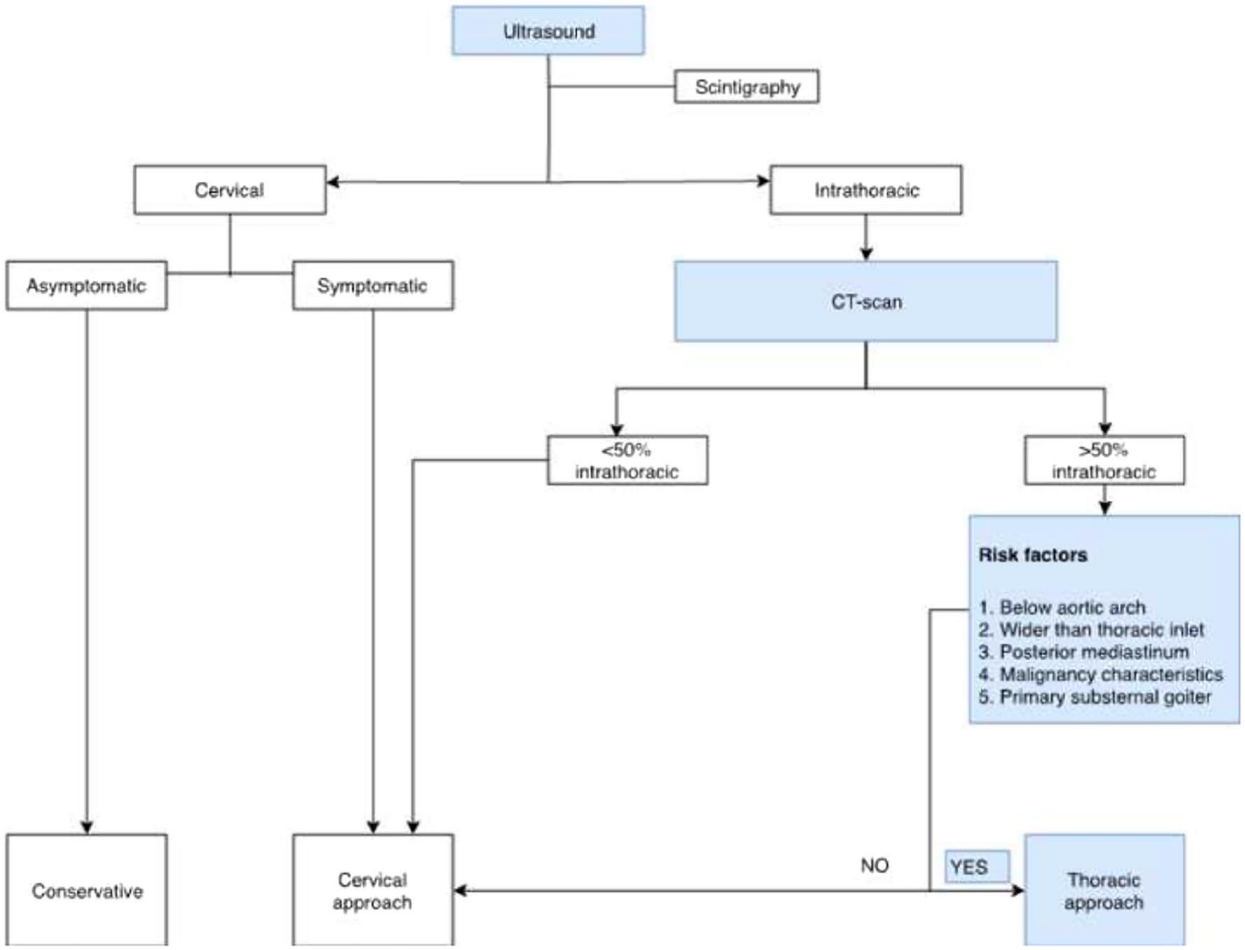
Flowchart for the decision-making process

In 84% of all intrathoracic goiters, surgery through cervicotomy was feasible to remove all thyroid tissue. Our number of people treated by cervicosternotomy seems quite high. This could be related to consistently following the flowchart (Fig. 3) and the fact of being a tertiary referral centre. Immediate morbidity was comparable to a standard thyroidectomy, only transient RLN palsy occurred more after cervicosternotomy. There was no increased risk of permanent hypocalcemia, permanent RLN palsy or reinterventions for bleeding, despite the increased complexity of surgery as shown by increased duration of surgery, blood loss and weight of the specimen. No patients in the sternotomy group developed a keloid scar or excessive scar formation. Remarkably, the incidence of transient hypocalcemia was higher in the standard thyroidectomy group, when comparing to surgery for intrathoracic goiters. This standard thyroidectomy group mainly includes surgery for Graves’ disease and thyroid cancer with central neck dissection, both known risk factors for hypoparathyroidism. The absent permanent hypocalcemia in all patients with intrathoracic goiter could be due to the low number of cases, the extensive experience of the surgeon or a surgical bias. In cases with retrosternal goiter, it is of utmost importance to not harm the upper 2 parathyroid glands, because of the high risk of not viewing or damaging the blood supply of the lower parathyroid glands.

The major strengths of this study are the standardized pre-operative preparation, surgical procedure and post-operative follow-up, and the large series of patients operated by a highly experienced endocrine surgeon. In addition, detailed information has been collected on demographics and disease-related characteristics, that could confound the risk of the major complications.

The authors acknowledge there may be residual confounding variables and only a small set of confounders was used because of limited power. However, despite a different baseline situation and an a-priori higher risk of poorer outcome, the differences between the groups were still relatively limited for most outcomes.

According to our experience, surgery for an intrathoracic goiter is especially challenging if the goiter is shaped like an apple and/or crosses the midline of the mediastinum. Previous studies also reported an iceberg-shaped goiter or a pure intrathoracic goiter as an independent risk factor for an extra cervical approach [15, 16].

The preoperative decision to use a thoracic approach entails interesting advantages. First of all, the patient can be prepared and informed about the procedure and possible specific risks. Second, the surgeon can be optimally prepared if the decision about the approach is made in advance. Third, the assistance of a thoracic surgeon (if the endocrine surgeon lacks skill to perform a sternotomy and thoracic dissection) and the specific material can be provided. Fourth, the anesthesiologist can make a more accurate preoperative evaluation of the patient and his perioperative needs. Moreover, when a thoracic approach is indicated and a cervical approach is applied instead, the risk of complications may increase. In specific, the performance of digital manipulations to retract the intrathoracic part of the goiter will increase the risk of incomplete removal and morcellation of the thyroid resulting in a “forgotten” goiter [1]. This is mediastinal thyroid tissue remaining after a so-called “total” thyroidectomy. Another risk of morcellation is soiling in the case of a malignant goiter. Performing a thoracic approach can reduce the risk of a traumatic hemorrhage, because the surgeon is not dissecting blindly in the area of the great vessels [1, 2, 7, 16]. Additionally, there are arguments for a reduced risk of RLN palsy and hypoparathyroidism in a thoracic approach compared to digitally cervical retraction [4, 7]. More blood loss during surgery and an increased hospital stay have been reported for cervicosternotomy compared to cervical approaches [2]. This was confirmed in the present study. This highlights the increased complexity of the surgical procedure when adding a sternotomy. However, cervicosternotomy does not lead to an excessive risk of major complications including RLN palsy and hypocalcemia, besides an increased risk of pulmonary complications [4, 7, 9, 15, 17]. No reintervention for bleeding was noted in the cervicosternotomy group in our series. It is a fact that mediastinal vessel exposure, mediastinal vessel ligation and intraoperative bleeding control from the mediastinum are better with sternotomy compared to the ‘blind’, manual dissection in case of cervicotomy.

Understanding the benefits and disadvantages and determining the probability of a thoracic approach still remain the biggest challenges. Every preoperative planning for thyroid surgery, either cervical or thoracic, requires an ultrasound. A supplementary scintigraphy can be carried out if the thyroid-stimulating hormone is low or suppressed. If the ultrasound indicates a risk of a retrosternal goiter or when the border of the lower pole can’t be felt on clinical examination, a neck and chest CT should be done to evaluate its size and location in relation to the surrounding structures [1–4].

Because the more invasive nature does not translate into more associated morbidity, recent literature suggests that a thoracic approach should be considered more often in the preoperative phase [2, 18]. Not every goiter reaching below the aortic arch needs a thoracic approach. It must be emphasized that there is no reason to avoid the use of this type of access, keeping in mind the benefits, or the risk of forgotten goiters in intrathoracic goiter which would need an invasive procedure afterwards anyway [1, 5, 18].

Based on this information and our own experience, we created a flow chart, which can guide surgeons in the preoperative decision-making process for dealing with multi-nodular goiters (Fig. 3). The algorithm shows that in the case of an asymptomatic cervical goiter, a conservative approach is recommended. In case of a substernal goiter, a CT-scan should be performed, and surgery discussed with the patient to prevent future complications. In most cases, a full sternotomy is preferred as it provides excellent visualization of the goiter, mediastinum and related structures [4]. In some cases, a less invasive approach can be sufficient: sternal split, lateral thoracotomy and posterolateral thoracotomy are described previously [1, 4, 19].

In conclusion, this study shows that despite its invasiveness, surgery for goiters with retrosternal extension is safe without an excessive risk of major complications. Reintervention was higher in the cervicotomy group compared to the cervicosternotomy group. An extensive standardized pre-operative preparation, a CT-scan to determine the extent and complexity and multidisciplinary approach are crucial in surgical decision making.

## Supplementary Information

Below is the link to the electronic supplementary material.

Supplementary file1 (DOCX 46 KB)

## Declarations

### Availability of data and material

Although the data are anonymised, these can not be shared open access because of the detailed information on all patients. Upon reasonable request (and necessary approval of the ethics committee), the corresponding author can be contacted for potential access to the data.
