# Supplementary figures and images for: Combined cervicosternotomy and cervicotomy for true retrosternal goiters: a surgical cohort study
Source: Updates Surg. 2021 Mar 29;73(4):1–10. doi: 10.1007/s13304-021-01027-1 (PMC8397680; doi:10.1007/s13304-021-01027-1)

**Supplementary Figure 1: Flowchart of the Study Cohort**

**
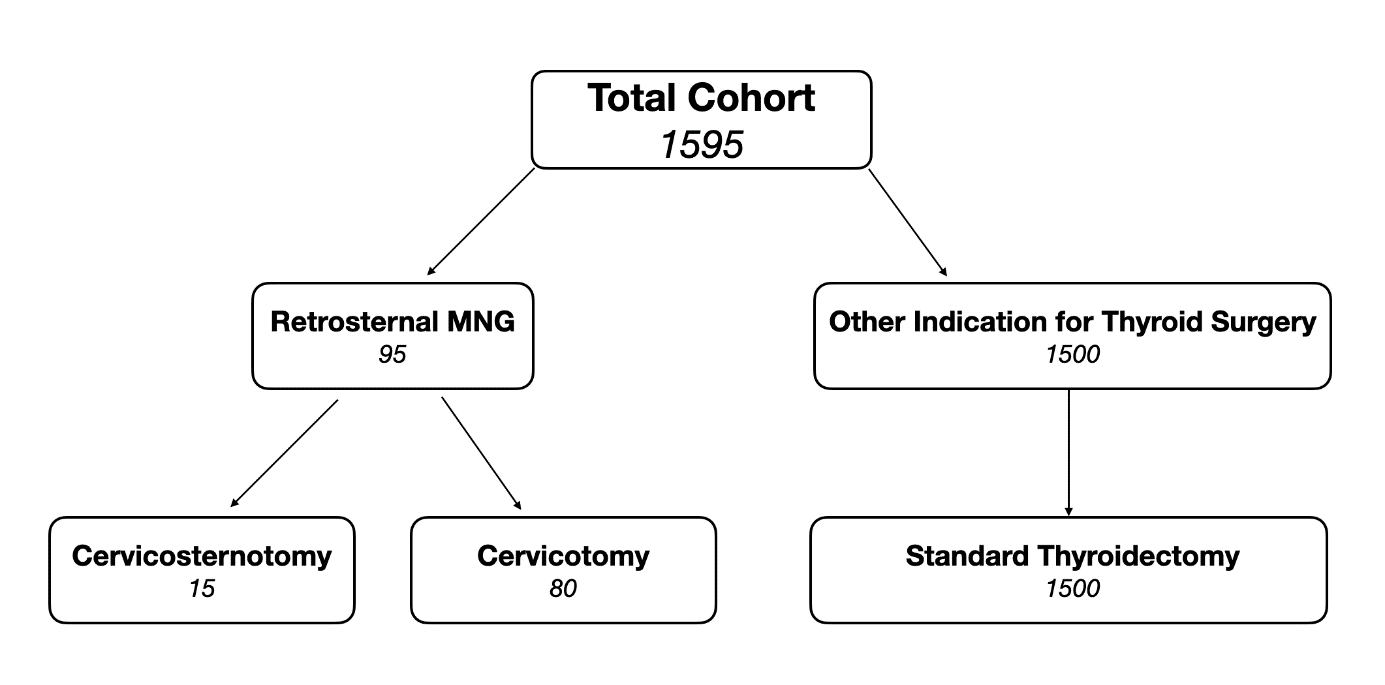
**

Supplement: Supplementary file 1 — Supplementary file1 (DOCX 46 KB) [file 13304_2021_1027_MOESM1_ESM.docx]
